# The application of a medium-chain fatty diet and enteral nutrition in post-operative chylous leakage: analysis of 63 patients

**DOI:** 10.3389/fnut.2023.1128864

**Published:** 2023-07-20

**Authors:** Ke Wang, Jiaming Xiao, Li Li, Xu Li, Yilun Yang, Zhiyu Liu, Jing Jiang

**Affiliations:** ^1^Department of Clinical Nutrition, Second Affiliated Hospital of Dalian Medical University, Dalian, Liaoning, China; ^2^Department of Nutrition and Food Hygiene, School of Public Health, Dalian Medical University, Dalian, Liaoning, China; ^3^Department of Urological Surgery, Second Affiliated Hospital of Dalian Medical University, Dalian, Liaoning, China; ^4^Department of Nursing, Second Affiliated Hospital of Dalian Medical University, Dalian, Liaoning, China

**Keywords:** chylous leakage, nutritional intervention, medium-chain fatty diet, enteral nutrition, post-operation

## Abstract

**Background:**

Post-operative chylous leakage (CL) is the pathologic leakage of chylomicron fluid after surgery. This retrospective study was performed to evaluate a uniform oral nutrition management strategy on the post-operative CL.

**Methods:**

We retrospectively reviewed patients who developed post-operative CL and received consultation from a clinical nutritionist in seven departments of the Second Affiliated Hospital of Dalian Medical University from May 2020 to April 2022. We designed the oral nutrition intervention program which mainly standardized the type and amount of foods contained in the medium-chain triglyceride (MCT) diet. The influencing factors of curative efficacy were analyzed. Finally, binary logistic regression analysis was conducted to observe the relationship between curative efficacy and potentially predictive variables, including post-operative albumin, post-operative hemoglobin, surgical procedure, and drainage volume at consultation.

**Results:**

Sixty-three patients with post-operative CL were included in this analysis. Of this number, 58 patients were cured successfully without other treatments. Three patients had a significantly prolonged recovery period, and the remaining two cases were treated by reoperation therapy. The leakage volume at the initiation of enteral intervention had no statistically significant difference in seven surgical departments and surgical sites (left, right, median, and bilateral). The length of stay (LOS) of patients with CL after the intervention was not significantly increased in cardiac, hepatobiliary, gastrointestinal, and urological surgeries. Patients with CL had longer LOS than those without CL in gynecology (*P*=0.044) and thyroid surgery departments (*P*=0.008). Each unit increase in post-operative hemoglobin would increase the probability of an effective outcome by 8%, which was statistically significant (*P* = 0.037).

**Conclusion:**

In treating patients with post-operative CL, we recommend the MCT diet and EN as the first option, rather than fasting, parenteral nutrition (PN), or octreotide.

## 1. Introduction

Chylous leakage (CL) is a pathological status in which chylomicron fluid leaks from the lymphatic vessels (1). CL is commonly associated with surgical trauma, abdominal malignancies, cirrhosis, and infection (2–4). In clinical settings, CL occurs mostly in patients after surgical trauma, and the incidence of CL is higher in tumor resection with lymphadenectomy (5). The incidence of CL after thyroid surgery is approximately 0.5–1.4% (6, 7). The incidence of CL after general thoracic surgery ranges from 0.4% to 3.9% (8, 9). CL may occur in 1–16% of patients after pancreatectomy (10, 11). CL occurs in ~3.8%−5.1% of nephrectomy (12, 13) and approximately 0.3–7.4% in gynecology (14, 15). Patients with post-operative CL are often in a state of high energy expenditure and protein requirements due to traumatic stress. CL may lead to decreased blood volume, malnutrition, and a compromised immune system (16), severely affecting post-operative recovery, prolonging the length of stay (LOS), and even increasing mortality (17, 18). Four main clinical treatments are available: medium-chain triglyceride diet (MCT diet) [with or without enteral nutrition (EN)] or low-fat diet, parenteral nutrition (PN), drug therapy (somatostatin such as octreotide), and surgery (4, 19–21). However, there is no consensus on the optimal management of CL (22).

CL also lacks uniformity for a definitive diagnosis (17, 23–25). Generally, drainage fluid triglyceride levels >110 mg/dl may indicate the possibility of CL (26). Long-chain triglycerides (LCTs) are broken down in the intestine into chylomicrons, which enter the circulation through the lymphatics. Lymphatic vessels transporting chylomicrons form an elongated lymphatic structure with saccular dilatation called the cisterna chyli (CC) anterior to the l1–l2 vertebrae (24, 27). All lymphatic vessels together form the thoracic duct (TD), except for the lymphatic vessels in the right upper trunk and right upper extremity (15). Triglycerides from the lymphatic vessels leak into the abdominal cavity to form chylous ascites (CA) (28). Collection in the pleural cavity results in chylothorax (24). Chyluria disease (CD) can be considered when a fistulous communication between the lymphatic trunk or lymphatic vessels and the urinary tract contributes to intermittent or continuous milky white urine (29, 30) because of various causes. CL can occur when the TD, CC, and lymphatic trunk or lymphatic vessels around the intestine are blocked or ruptured (31, 32). CL is distinguished from lymphatic leakage by the pathological leakage of chylomicron rather than pure lymphocytes from broken lymphatic vessels after digestion and absorption through the gastrointestinal tract. Therefore, drainage fluid appears usually milky white or even pink (2, 33). Whereas, short- and medium-chain triglycerides (MCTs) enter the liver directly through the hepatic portal vein (34). A more direct basis for confirmation is that the fluid becomes clear after the cessation of the LCT diet (35).

The intervention of an MCT diet could not only meet patients' nutritional needs and reduce their discomfort but also reduce chylomicron and promote lymphatic vessel healing (36, 37). By observing the outcomes of previous dietary interventions in patients with CL, we found that by severely restricting the intake of LCT, the drainage fluid would immediately become clear. Conversely, it would quickly become a turbid liquid. In the present study, 63 patients who underwent thyroid surgery, cardiac surgery, thoracic surgery, hepatobiliary surgery, gastrointestinal surgery, and surgeries from urology and gynecology and had post-operative CL were evaluated retrospectively. We screened patients who underwent the same surgery during the same period as these CL patients did not develop CL. LOS between CL and non-CL patients was compared to discuss the effect of this nutritional management strategy. We also explore the factors that influence the effectiveness of this management strategy.

## 2. Methods

### 2.1. Patient selection and data collection

This study was approved by the ethics committee of our hospital. Certainly, all patients were given informed consent to the oral nutritional intervention they received. After the approval, we retrospectively collected and analyzed the data of patients with CL after surgery between March 2020 and April 2022.

**Figure 1:**
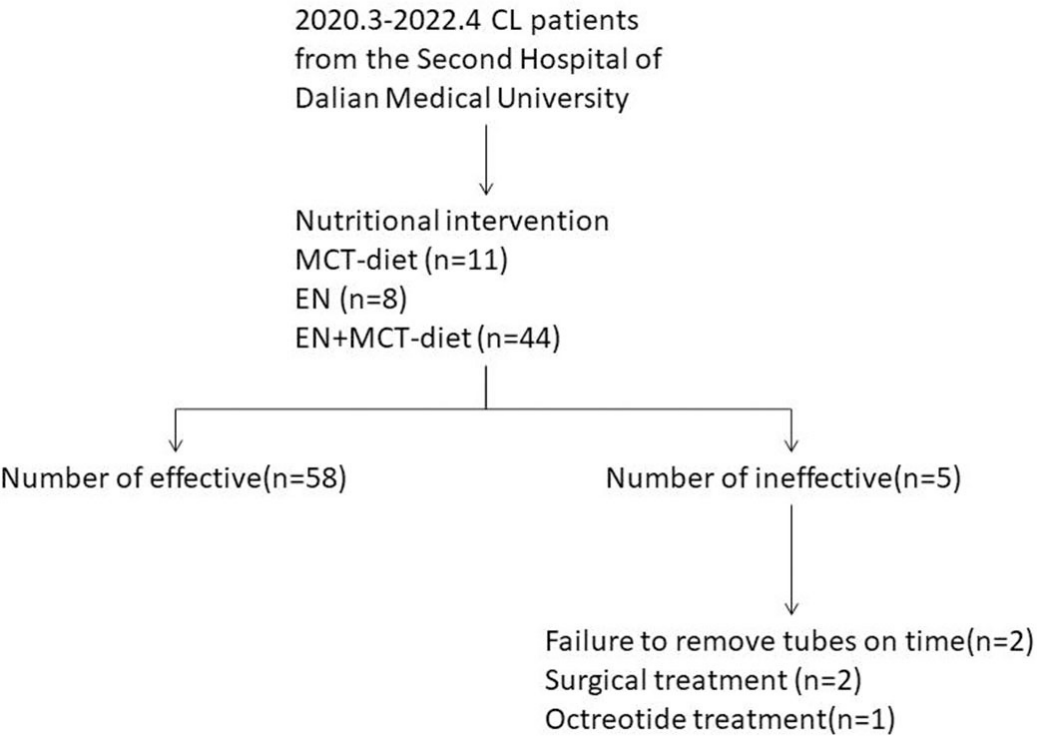
Flow diagram.

The study retrospectively reviewed patients who were provided nutritional intervention because of the post-operative presence of milky, murky, pinky, whitish, or yellowish drained fluid from March 2020 to April 2022 at the Second Affiliated Hospital of Dalian Medical University, People's Republic of China. Sixty-three CL patients who had undergone cardiac, thyroid, thoracic, hepatobiliary, gastrointestinal, urological, and gynecological surgeries were included (Figure 1). Clinical data were collected retrospectively from a review of electronic medical records. We collected patient data on demography, preoperative BMI, surgical procedure and site, post-operative nutrition-related laboratory results (albumin and hemoglobin), daily volume of CL, LOS, and days of CL. These patients were classified according to the surgical site (medial, left, right, and bilateral sites of the body) and compared statistical differences in the amount of drainage among the four sites of surgery. A logistical regression analysis was used to assess the association between potential risk factors (surgical procedure, volume at consultation, post-operative hemoglobin, and post-operative albumin) and efficacy.

### 2.2. Identification and management of CL

CL was clinically diagnosed after the observation of a milky, murky, pinky, whitish, or yellowish drainage fluid. In cases of suspected CL but unclear clinical features, drainage fluid triglyceride concentration was determined to differentiate between CL and lymphatic leak. A concentration of >1.2 mmol/L confirmed a diagnosis of CL. Not all such cases underwent fluid triglyceride concentration determination, but nutritional interventions were nonetheless performed.

### 2.3. Oral nutritional intervention program

The nutritional intervention program was provided by nutritionists from the Clinical Nutrition Department in our institution. Patients' energy requirements were calculated using the H–B equation, and protein requirements were set to 1.0–1.5 g/kg body weight according to patients' individual situations. The diet and enteral formula in our nutrition strategy were standardized. Briefly, we provided the MCT diet consisting of rice or steamed bread (containing 200 g of rice or flour), 500 g of vegetables, 6 egg whites, 6 g of salt, 250 ml of skim milk, and 25 g of coconut oil. The diet provides ~1,200 kcal of energy and ~45 g of protein. Fruits of the patient's choice (except those with high-fat content, such as durian and avocado) were usually allowed. All other foods were excluded. The taste and texture of this diet are similar to those of a normal Chinese diet. The diet was supplemented with low-fat EN (Yi di su, Hangzhou Nutrition Biotechnology Co., Ltd) and whey protein powder (Nutrasumma, Qingdao Nutrasumma Health Technology Co., Ltd) between meals when the dietary intake of protein and energy was inadequate. The MCT diet was given when the patient was able to eat a regular diet. A low-fat EN (Yi di su) and whey protein powder (Nutrasumma) were provided to meet the patient's energy and protein demands when only a liquid diet was available (Table 1). The drainage tubes were removed at the discretion of the surgeon, and ordinarily, a volume of <30–50 ml/24 h of liquid from the tube was an indication for tube removal. Patients continued the MCT diet for 1–2 weeks after the removal of the drainage tube before transitioning to normal meals. When fluid drained from the drainage tube does not reduce in volume after the oral diet for 3 consecutive days, patients were fasted off water and diet and administered PN or growth inhibitor drugs (octreotide). At the discretion of the surgeons or nutritionists, a second surgery procedure should be performed. If the tube is not removed by the time the patient is discharged, discharge instructions are given by the nutritionists from the Clinical Nutrition Department. Such patients would continue taking the MCT diet, whey protein powder, and fat-soluble vitamin supplements at home. Patients were provided with full diet recommendations on types and quantities of food allowed and foods to be excluded (Table 2).

**Table 1:** Carbohydrates, proteins (amino acids), and fats in the MCT diet and low-fat EN.

	**MCT diet**	**Low-fat formula**	**Whey Protein**
	**Provided by dietitian**	**Yi di su** ^®^	**Nutrasumma** ^®^
Carbohydrates	12.8	18.6	0
Proteins	3.75	4.3	25
Fats	2.08	0.43	0

Each value was expressed in g/100 kcal.

**Table 2:** Dietary instructions for patients with CL.

**Types of food**	**Our suggestion**
Staple foods	Use steaming and boiling cooking methods; avoid frying, deep-frying, and foods made with oil
Animal and seafoods	Egg whites are allowed; animal foods, such as egg yolks, fish, shrimp, and meat are not allowed
Dairy products	Skimmed milk and skimmed yogurt can be consumed; avoid whole milk and its products
Bean products	Not allowed to consume
Vegetable category	Allowed to consume
Fruit category	Aside from fruits with high-fat content, such as durian and avocado, all others can be consumed
Oils	Food should be cooked with coconut oil but not with animal or vegetable oils
Whey protein powder	Based on the patient's daily requirement minus the protein content of the MCT diet
Multivitamin tablets	One tablet a day

### 2.4. Statistical analysis

A normality test was performed for all continuous variables. Albumin levels before and after intervention were normal distribution, presented as mean ± SD. Other data were skewed distribution, presented as median (ranges). Categorical variables were compared using the χ^2^ test or Fisher's exact test. In comparing categorical data using Pearson's χ^2^ test, Fisher's exact test was used when the number of cells was <5. The Mann–Whitney U-test or variance analysis was used to compare multiple continuous independent samples. The Wilcoxon or Student's *t*-test was used to compare two independent samples. Additionally, binary regression analysis was used to evaluate the potential impact of interested predictors on the efficiency of intervention, and factors with a *p*-value of < 0.05 (including surgery procedure, leakage volume at the consultation, post-operative albumin, and post-operative hemoglobin) were included in logistic regression analysis. Statistical significance was defined as a *p*-value of <0.05. All statistical analyses were performed using the Statistical Package for Social Sciences version 25.0 (IBM Corp., Armonk, NY, USA).

## 3. Results

### 3.1. Demographic and baseline characteristics of patients

We collected data on all patients with CL who underwent oral nutritional intervention from March 2020 to April 2022. Of the 63 patients, 26 were men and 37 were women. Their age ranged from 31 to 83 years (median, 61), and their BMI ranged from 15 to 34 kg/m^2^ (median, 23.9). Surgical sites were divided into middle, left, right, and bilateral according to the location of the surgical site in the body. Location on the middle side was performed in 11 patients (17.5%), on the left side in 18 patients (28.6%), on the right side in 17 patients (27.0%), and on the bilateral sides in 17 patients (27.0%). Types of surgeries were thyroid surgery in 12 patients (19.0%), cardiovascular surgery in 6 patients (9.5%), thoracic surgery in 2 patients (3.2%), hepatobiliary surgery in 4 patients (6.3%), gastrointestinal surgery in 24 patients (38.1%), urological surgery in 5 patients (7.9%), and gynecological surgery in 10 patients (15.9%). These patients were classified according to surgical sites (medial, left, right, and bilateral sites) and compared with the leakage volume of onset of intervention among the four sites of surgery. The volume of fluid was 110 (67–1,660) (ml) for patients with a middle surgical site, 120 (20–500) (ml) for patients with a left-sided surgical site, 220 (44–510) (ml) for patients with a right-sided surgical site, and 95 (30–535) (ml) for patients with a bilateral site. No statistically significant difference in drainage volume among the four groups was recorded (*P* = 0.422). The leakage volume of onset of intervention was 195 (80–598) (ml) for patients with thyroid surgery, 525 (185–1,020) (ml) for patients with cardiovascular surgery, 1,070 (1,020–1,120) (ml) for patients with thoracic surgery, 625 (250–910) (ml) for patients with hepatobiliary surgery, 137 (34–630) (ml) for patients with gastrointestinal surgery, 610 (360–1,440) (ml) for patients with urological surgery, and 485 (160–1,000) (ml) for patients with gynecological surgery. In this study, among the surgery departments, there was a statistically significant difference in drainage volume among different types of surgeries (*P* < 0.05) (Table 3).

**Table 3:** Patients' demographics.

**Parameter**		**Median/*n***	**Range/%**
Age (yr)		61	31–83
Sex	Men	26	41.3
	Women	37	58.7
BMI (kg/m^2^)		23.9	15–34
Location of surgery^*^ (*n*)	Middle	11	17.5
	Left	18	28.6
	Right	17	27.0
	Bilateral	17	27.0
Types of surgeries (*n*)	Thyroid surgery	12	19.0
	Cardiac surgery	6	9.5
	Thoracic surgery	2	3.2
	Hepatobiliary surgery	4	6.3
	Gastrointestinal surgery	24	38.1
	Urological surgery	5	7.9
	Gynecological surgery	10	15.9
Leakage volume of onset of intervention (ml)			
Location of surgery^*^	Middle	110	67–1,660
	Left	120	20–500
	Right	220	44–510
	Bilateral	95	30–535
Types of surgeries#	Thyroid surgery	195	80–598
	Cardiac surgery	525	185–1,020
	Thoracic surgery	1,070	1,020–1,120
	Hepatobiliary surgery	625	250–910
	Gastrointestinal surgery	137	34–630
	Urological surgery	610	360–1,440
	Gynecological surgery	485	160–1,000

# The leakage volume of onset of intervention was P < 0.05 between different departments.

* It is divided into middle, left, right, and bilateral according to the location of the surgical site in the body.

### 3.2. Incidence and management of CL

Among all patients, 12 patients were from thyroid patients (CL incidence 3.72%), 6 patients were from cardiovascular surgical patients (CL incidence 5.22%), 4 patients were from hepatobiliary patients (CL incidence 2.25%), 24 patients were from gastrointestinal surgical patients (CL incidence 2.15%), 5 patients were from urological patients (CL incidence 1.28%), and 10 patients were from gynecologic surgical patients (CL incidence 2.77%).

**Figure 2:**
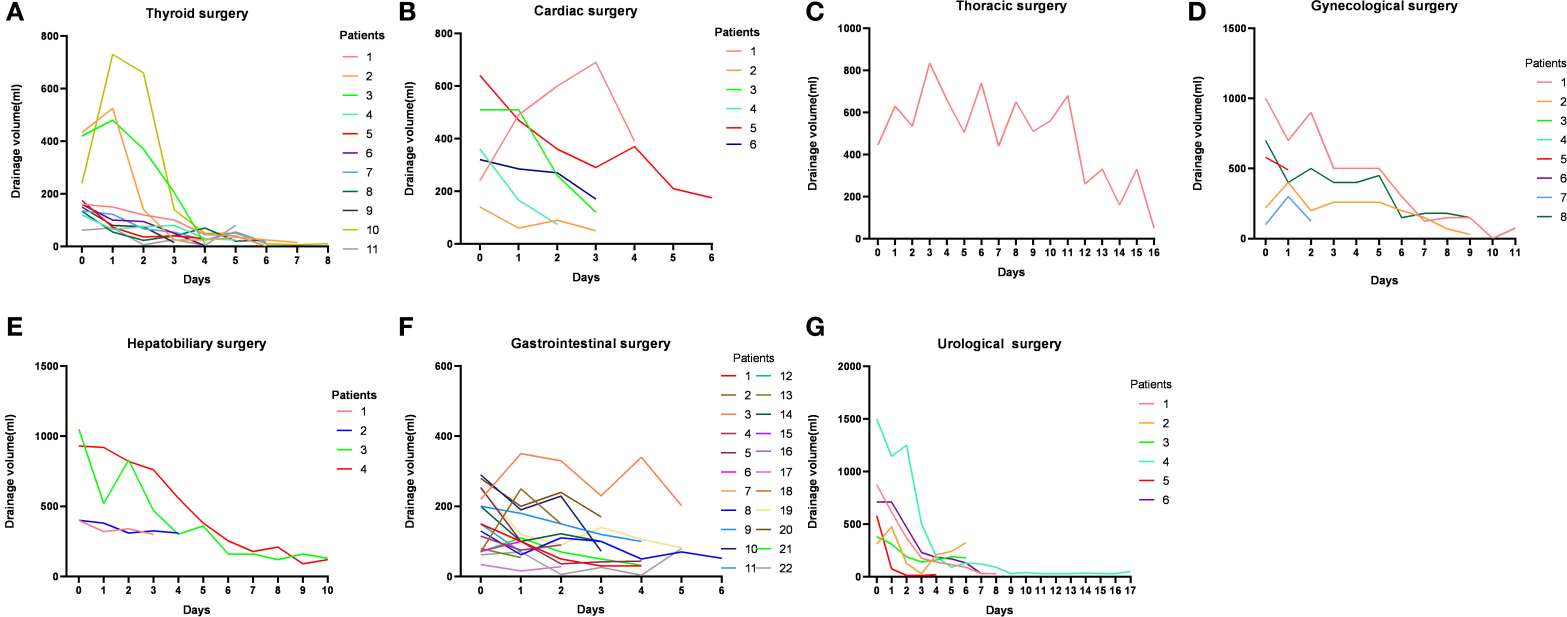
Daily changes in the volume of chylous leakage in successful cases in seven types of surgeries.

All patients had an initial oral nutritional intervention, 11 patients had an MCT diet, 44 patients had an EN plus MCT diet, and 8 patients had an EN. The chylous leakage volume began to decrease 1 day after starting the MCT and/or EN treatments in most of the seven types of surgeries cases. For most successful cases, the first 3 days of leakage volume tend to reduce rapidly (Figure 2). One case from thyroid surgery had leakage volume changed from 345 ml to 500 ml after 3 days with the MCT diet plus EN, so a secondary surgery was performed. One case from thoracic surgery had no significant change in leakage volume after 2 days with EN, but had leakage volume changed from 1,125 ml to 1,610 ml after fasting 5 days, so a secondary surgery was performed. Two cases from gastrointestinal surgery were discharged with a tube and at the first outpatient return visit still failed to be extubated. One case from gynecological surgery was readmitted for CL within 30 days.

### 3.3. Effectiveness of nutritional intervention

In this study, an effective oral nutritional intervention was defined as (1) tube removal before discharge; (2) tube was not extubated before discharge but extubated at the first outpatient return visit as scheduled; and (3) no readmission for CL within 30 days. An ineffective oral nutritional intervention was defined as (1) secondary surgery was implemented; (2) PN >50% energy; (3) use of octreotide; (4) discharged without extubation and the tube at the first outpatient return visit still failed to be extubated; and (5) readmission for CL within 30 days.

The number of effective cases after intervention was 11 (91.7%) for thyroid surgery, 6 (100%) for cardiovascular surgery, 1 (50%) for thoracic surgery, 4 (100%) for hepatobiliary surgery, 22 (91.7%) for gastrointestinal surgery, 5 (100%) for urological surgery, and 9 (90%) for gynecological surgery, amounting to 58 (92.1%) cases in total. No statistical differences in effective rates were observed among departments (*X*^2^ = 4.7, *P* = 0.533) (Table 4).

**Table 4:** Comparison of efficiency after intervention in different types of surgeries.

**Types of surgeries**	**Number of effective cases [*****n*** **(%)]**		***X*^2^ test**	**Days of drainage [Median (ranges)]**		**K–W test**	**LOS [Median (ranges)]**		**Wilcoxon test**
	**Effective**	**Non-effective**	***P-*** **value**	**Effective**	**Non-effective**	***P-*** **value**	**CL**	**Non-CL** ^*^	***P-*** **value**
Thyroid surgery	11 (91.7)	1 (8.3)	0.533	6 (4–8)		0.008	14 (10–21)	11 (7–31)	0.008
Cardiac surgery	6 (100)	0 (0)		4 (3–7)	0		15 (11–28)	11 (5–28)	0.321
Thoracic surgery	1 (50)	1 (50)		17	6		6	7 (5–12)	-
Hepatobiliary surgery	4 (100)	0 (0)		8 (4–11)	-		21 (16–23)	14 (1–15)	0.148
Gastrointestinal surgery	22 (91.7)	2 (8.3)		3 (1–7)	30		14 (8–25)	13 (11–31)	0.555
Urological surgery	5 (100)	0 (0)		7 (1–18)	-		14 (9–19)	9 (5–13)	0.148
Gynecological surgery	9 (90)	1 (10)		3 (1–21)	48		15 (9–36)	11 (8–27)	0.004
Total	58 (92.1)	5 (7.9)							

* Non-CL, patients who underwent the same surgery procedure at the same period but did not develop CL.

M, median; MD, median difference; *p*-value < 0.05.

We recorded the days of drainage for all patients when they were in the hospital, but those who were discharged with a drainage tube and were extubated at the first outpatient return visit as scheduled would usually be considered a better recovery at discharge and after discharge. Both the days and volume of drainage after discharge could not be followed up. For the other five patients on whom oral nutritional therapy failed to work, they continued to receive other medical treatments due to high drainage volume. Therefore, the drainage volume and days of these patients were recorded in the inpatient medical records. Days of drainage in effective patients were as follows: 6 (4–8) days in thyroid surgery, 4 (3–7) days in cardiac surgery, 17 days in thoracic surgery, 8 (4–11) days in hepatobiliary surgery, 3 (1–7) days in gastrointestinal surgery, 7 (1–18) days in urological surgery, and 3 (1–21) days in gynecological surgery. Days of drainage in non-effective patients were as follows: 6 days in thyroid surgery, 6 days in thoracic surgery, 30 days in gastrointestinal surgery, and 48 days in gynecological surgery (Table 4).

The LOS was 14 (10–21) days in patients with CL from the thyroid surgery department and 11 (7–31) days among patients without CL, with a statistically significant difference in LOS between the two groups (Z = 2.6, *P* = 0.008). Patients with CL stayed longer than those without CL in the thyroid surgery department after the nutritional intervention. The LOS was 15 (9–36) days in patients with CL from the gynecology department and 11 (8–27) days in patients without CL, with a statistically significant difference in LOS between both groups (Z = 2.0, *P* = 0.044). Patients with CL stayed longer than those without CL in the gynecology department after the nutritional intervention. The LOS of patients with CL after the nutritional intervention in the other departments did not differ from those of patients without CL (Table 4). Five of the included patients had an ineffective intervention; one patient resorted to octreotide use, two patients were not extubated as scheduled, and two patients underwent secondary surgery.

### 3.4. Factors associated with recovery after intervention in post-operative CL

A logistic regression model was established by including post-operative albumin, post-operative hemoglobin, surgical procedure, and drainage volume at consultation (Table 5). The relationship between post-operative albumin and intervention outcome was not statistically significant (*P* = 0.191). The relationship between surgical procedures and intervention outcomes was not statistically significant (*P* = 0.837). Additionally, the volume of consultation on intervention outcomes was not statistically significant (*P* = 0.995). Each unit increase in post-operative hemoglobin would increase the probability of an effective outcome by 8%, which was statistically significant (*P* = 0.037).

**Table 5:** Multivariate analysis of factors associated with recovery after intervention in post-operative CL.

	**B**	**S.E**.	**Wald**	**df**	**Sig**.	**Exp (B)**	**95% CI**	
							**Lower**	**Upper**
Procedure^*^	−0.237	1.153	0.042	1	0.837	0.789	0.082	7.563
Volume at consultation	0.000	0.002	0.000	1	0.995	1.000	0.995	1.005
Post-operative albumin	−0.220	0.168	1.713	1	0.191	0.803	0.578	1.115
Post-operative hemoglobin	0.077	0.037	4.334	1	0.037	1.080	1.004	1.160
Constant	2.693	5.862	0.211	1	0.646	14.780		

* neck = 1; thorax = 2; abdomen = 3; pelvis = 4.

### 3.5. Albumin levels before and after the intervention and variation of leakage volume for different interventions

Consideration of the potential serious influence of CL on patients' nutrition conditions, we observed albumin level changes, too. The analysis of 58 successful patients' albumin levels before and after intervention revealed that the value after the intervention was higher than that before the intervention (*P* = 0.007). Finally, whether different oral nutritional interventions exert differences in CL outcomes aroused our interest. We divided our oral nutritional intervention method into three groups: MCT diet, EN, and MCT diet plus EN, and differences in albumin before and after intervention among the three groups were observed. No significant differences in albumin level were recorded for different interventions (*P* = 0.555) (Table 6). Similarly, variations in drainage volume before and after consultation were analyzed; and no differences in median drainage volume among the three groups were recorded (*P* = 0.347) (Table 6). The intervention strategy did not compromise but improved, the nutritional status. There was also no difference in the impacts of the three different interventions on patient outcomes.

**Table 6:** Comparison of preintervention and postintervention albumin levels for different interventions and variations of leakage volume for different interventions.

**Intervention**	*N* ^*^	**Albumin (mg/L) (Mean** ±**SD)**		***F***	***P***	***N***	**Changes in leakage volume (ml/day) [Median (range)]**	***H***	***P***
		**Before intervention**	**After intervention**						
EN	6	33.09 ± 3.96	30.95 ± 6.70	0.596	0.555	8	50 (−15–590)	2.119	0.347
MCT diet and EN	32	32.27 ± 4.42	34.99 ± 5.17			44	105 (−75–1370)		
MCT diet	9	33.12 ± 2.62	34.29 ± 6.70			11	256 (30–475)		
Total	47	32.54 ± 4.03	35.11 ± 4.95						
*t*		−2.762						
*P*		0.07						

* Since albumin levels were not tested in 16 patients in the hospital; these cases were excluded. Hence, the albumin level in 47 patients was analyzed before and after the intervention. The ANOVA and *t*-test were used for statistical analyses.

## 4. Discussion

Post-operative CL is a surgical complication due to damage to the lymphatic system (38). Its occurrence as a surgical complication may induce malnutrition and lead to a state of immune compromise, even affecting the long-term outcome of patients undergoing surgical treatment for malignant disease (10, 39). The use of a dietary program such as the MCT diet in patients with CL can reduce the burden on damaged lymphatic vessels for the reason that MCT is not usually absorbed through the intestinal lymphatics (10). Since short- and medium-chain triglyceride acids are mostly water-soluble and absorbed through the portal circulation rather than gastrointestinal lymph, the MCT diet can bypass the gastrointestinal lymphatic system, allowing reduced chylous flow at the CL site, thus resulting in quicker healing. However, no unanimous consensus on its use has been reached (23, 40, 41). Octreotide can reduce visceral blood flow, lymphatic flow, and the secretion of digestive glands, thus lessening the absorption of LCT which decreases the triglyceride content of lymphatic vessels and supports the healing of the site where CL occurs quickly (42–44). TPN provides nutrients through the veins, thus avoiding the absorption of LCT into the lymphatic vessels through the intestine. However, long-term PN would weaken the intestinal functional and have adverse effect on the patient's management. TPN and/or octreotide are considered in cases where EN/diet is not effective (45, 46). Secondary procedures, such as lymphatic embolization, surgical ligation, and abdominal venous shunts, are mostly used in refractory cases after the failure of conservative treatment (10, 47). The MCT diet is the least invasive and most economical option. However, the scope of MCT diet use for CL is still unclear. This study sought to investigate the effect of the MCT diet and/or EN interventions on the prognosis of patients with post-operative CL by reviewing such interventions in patients with CL across different departments in the hospital from March 2020 to April 2022. Our study found that the MCT diet and EN had a positive effect on CL and were applicable to the vast majority of patients. Additionally, they could alleviate patients' discomfort and fear of fasting or secondary surgery. Therefore, for patients with post-operative CL, we recommend the MCT diet as a first line of intervention.

There was no report on the relationship between the maximum leakage and the necessity to initiate dietary interventions. Thyroid surgery specialists suggested criteria for secondary surgical treatment range from outputs of >500 mL/day to >1,000 mL/day output for 5 days (16). The published report suggests the conclusion. We thus conclude that drainage of more than 11.6 ml/kg of body weight per day predicted failure of conservative therapy and the volume might prove useful in guiding early thoracic duct ligation. For example, a person weighs 70 kg. Then, when his daily drainage volume reaches 812 ml, it indicates the failure of conservative therapy (48). The research about chylothorax suggested: early surgical intervention was indicated if drainage of >500 mL of chylous fluid was observed during the first 24 h. If the drainage was <300 ml/day 3 days after the chylothorax diagnosis, the patients continued the low-fat diet. If the volume remained >300 mL/day after 3 days, surgical intervention was considered (49). We observed the intervention effect in CL patients whose highest leakage volume after intervention onset was higher than 500 ml in this study. There were 10 cases ranging from 500 ml to 999 ml, of which three cases failed (1 case from thyroid surgery, 1 case from gastrointestinal surgery, and 1 case from gynecological surgery) and 7 cases succeeded (2 cases from thyroid surgery, 2 cases from cardiovascular surgery, 1 case from hepatobiliary surgery, 1 case from gastrointestinal surgery, and 1 case from gynecological surgery). There were six cases ranging from 1,000 ml to 1,999 ml, all of which were successful (one case from cardiovascular surgery, one case from thoracic surgery, one case from hepatobiliary surgery, two cases from urological surgery, and one case from gynecological surgery). There was one case of >2,000 ml that failed (from thoracic surgery). We found that the conservative treatment was still effective for chylous drainage of >500 ml/day (13 in 17 cases were successful) in the present study. Based on the results of these retrospective cases, we suggested that oral nutritional intervention should be tried as prior management. More active treatment should be adopted if there is no significant drainage output decrease within 1 to 3 days. The reason we made the suggestion is that in successful cases of this study, we observed an obvious drainage fluid reduction in volume and the fluid turning to clear approximately 3 days after the MCT diet and EN intervention, which was consistent with published data that reported success of fasting and octreotide treatment (49, 50). In our successful cases, the maximum drainage volume was 1,440 ml. Drainage volume should not be the only factor for the determination of the choice of treatment. Treatment effects can often be measured by how much volume changes in response to a particular intervention (16). Therefore, we believe that the MCT diet and EN can still be attempted for at least 3 days even if early drainage is within 1,500 ml.

The clinical efficacy of MCT and/or EN in this study was higher than the published literature. All other departments except thoracic surgery had an over 90% effective rate of intervention, and no statistical difference in effective rate was observed among different departments, suggesting that our nutritional intervention program was not affected by the different surgical procedures. The effective rates of conservative therapy in other studies were lower than in our study. A retrospective study about non-surgery CL reported that 66% of patients failed after conservative treatment (51). A systematic review indicated that the success rates of three non-surgical treatments with MCT diet, low-fat diet, and enteral nutrition were 77.3%, 75.9%, and 63.2%, respectively (36). In a collective review, only 43% of patients resolved CL with conservative treatment with the MCT diet alone (52). In a retrospective observational study of patients with CA following lymph node dissection due to gynecologic tumor, 5 patients (41.7%) had complete symptom relief with the MCT diet alone, while 7 (58.3%) patients underwent paracentesis with drainage of fluid (53). In another retrospective observational study, 71% of gynecological surgery post-operative CL patients responded to conservative treatment (28). The phenomenon, the higher efficiency rate in this study than aforementioned published reports, may be contributed that there is no consensus on the standardized diagnosis and therapy procedure of CL and its effective interventions (5). For this reason, here instead of confirming the diagnosis of CL based absolutely on drainage triglyceride level and drainage volume as advised in previous reports, we confirmed the diagnosis immediately upon the observation of cloudy drainage fluid after related surgery (lymph node dissection, especially, and abdominal aortic lymph node dissection) and administered nutritional interventions. Therefore, it is possible to advance the intervention time, which may result in a better outcome than previous studies (54, 55). In addition, the MCT diet in our hospital and dietary guidance at discharge are standardized, which has not been reported in other MCT diet-related studies. Standardized diet preparation and guidance could be more conducive to the accurate implementation of nutrition programs, thus ensuring effectiveness.

Previous studies noted longer LOS in CL patients than non-CL patients after colorectal cancer and pancreaticoduodenectomy (56, 57). In this study, the LOS for patients with CL was longer than that for those without CL in thyroid surgery and gynecology, too. The difference in LOS among patients in the thyroid surgery department [14 (10–21) days vs. 11 (7–13) days, *P* = 0.008] may be because cervical lymph nodes collect most of the lymphatic flow in the extremities and the trunk (58). The MCT diet had a greater effect on the reduction of chylous flow in the digestive tract and did not stop lymphatic flow production. Hence, although CL was better controlled in patients with CL after thyroid surgery, lymph leaks may be less likely to heal relative to other sites of surgery (33). This may explain the longer LOS for patients with CL after thyroid surgery. Additionally, patients with CL after gynecological surgery also had a longer LOS, [15 (9–36) days vs. 11 (8–27) days, *P* = 0.044]. In this study, all CL patients in gynecology had tumor occupation and underwent pelvic lymph node dissection, and most of the patients also underwent abdominal aortic lymph node dissection. We speculate that pelvic tumor resection leads to less compression around the lymphatic vessels, which is detrimental to the healing of the lymphatic vessels. While no statistical difference in LOS was observed between patients with CL and non-CL in other types of surgeries. For this outcome, we believe that the MCT diet standardized protocol allows for better home management, and we also have detailed guidance at discharge, so that patients can be discharged with the drain tube. Generally, the drain tube would be removed in the outpatient return visit 1 or 2 weeks later. Therefore, LOS is not significantly longer in patients with CL. Thus, hospital medical resources are saved and patients' subjective comfort is improved.

Days of drainage after intervention onset were also a significant parameter to evaluate the clinical effect. We compared the intervention effects of EN, MCT, and TPN. The results showed that the days of drainage was similar between the present study and the published study [review (59) and (60)]. For example, a systematic review showed that the median for days of drainage was 5, 7.5, 29, or 19 days in patients with chylous ascites cured by TPN alone after hepatobiliary surgery, gastrointestinal surgery, and urological surgery (59). In the present study, the duration median was 8, 3, or 7 days in hepatobiliary surgery, gastrointestinal surgery, and urological surgery. For gynecological surgery patients with chylous leakage cured by TPN alone, the median was 15 (7–16) days (60). The median for days of drainage was 3 (1–21) days (3 cases discharged with a drainage tube on day 1 of intervention and 1 on day 2) in the present study under a similar condition.

Additionally, we performed a binary logistic regression analysis of our nutritional intervention outcomes, which showed no statistically significant difference in the effect of post-operative albumin, surgical procedures, and volume of leakage on effective outcomes. What needs to be explained is that the thyroid surgery case was not included in the binary logistic regression analysis as post-operative albumin and hemoglobin levels were not determined among patients who underwent thyroid surgery. Higher hemoglobin levels on the first day after surgery had a promotive effect on the probability of effective intervention. We found no relevant reports on the analysis of factors associated with effective postintervention outcomes. However, it was reported that lower hemoglobin is associated with a higher incidence rate of CL (14), which may predict that lower hemoglobin probably influences the effects of oral nutritional interventions. Our analysis of post-operative hemoglobin on intervention outcomes may inform future studies.

Limitations of this study are as follows: (1) This study was a single-center retrospective analysis, and no comparison could be made with other hospitals. (2) We also did not compare the efficacy of our intervention with other interventions (fasting, octreotide, and secondary surgery), which may make the results less convincing. The reason is that on the one hand, we used oral nutrition intervention in the initial stage of clinical intervention, and it achieved a good effect. Therefore, we adopted this method for all patients. On the other hand, before we regulated oral interventions, this phenomenon was not well-documented in our medical documentation, making it difficult to retrieve these cases from our medical record system as historical controls. (3) Some clinical data (e.g., post-operative albumin and drained triglycerides) were incomplete, as they were not tested in some patients at that time. As a result, it is difficult to draw strong conclusions about the effectiveness of our approach. (4) Although we have compared efficacy with published studies, there is a problem of inconsistent baseline. (5) There may also be some reasons leading to different discharges and extubation indications in different departments, thus affecting the statistical results of discharge time. As for the strength of this study, conservative treatment, such as MCT diet and EN, achieved successful outcomes while alleviating patient discomfort, fear of medication and secondary surgery, and reduced complications without significantly prolonged LOS. Although CL is a relatively rare clinical complication, it can seriously affect patients' prognosis. Prospective clinical intervention trials could be conducted to explore the mechanisms of treatment with MCT diet/EN of CL. In addition, the role of hemoglobin in the MCT treatment of CL can be explored.

## 5. Conclusion

In conclusion, ~90% of post-operative CL cases could be cured with our MCT diet/EN management strategy, which is higher than what was reported in many studies. Furthermore, the post-operative hemoglobin level can promote the prognosis of patients with CL. We believe that this management strategy works well, with a minimal patient burden and good nutritional condition. We detail this strategy in the study, hoping to provide useful information to others.

## Data availability statement

The raw data supporting the conclusions of this article will be made available by the authors, without undue reservation.

## Ethics statement

Written informed consent was obtained from the individual(s) for the publication of any potentially identifiable images or data included in this article.

## Author contributions

ZL designed the research and revised the manuscript. JJ collected the data of all cases. KW developed a treatment plan and wrote the manuscript. JX analyzed the data. LL, XL, and YY performed clinical nutrition therapy. All authors contributed to the manuscript and approved the submitted version.
